# The STAR collaborative nonsuicidal self-injury study: methods and sample description of the face-to-face sample

**DOI:** 10.1186/s13034-024-00820-1

**Published:** 2024-10-30

**Authors:** Jennifer Spohrs, Anna Michelsen, Birgit Abler, Andreas G. Chioccheti, Ulrich W. Ebner Priemer, Jörg M. Fegert, Saskia Höper, Tina In-Albon, Michael Kaess, Michael Koelch, Elisa Koenig, Julian Koenig, Laura Kraus, Sandra Nickel, Philip Santangelo, Christian Schmahl, Maurizio Sicorello, Patrice van der Venne, Paul L. Plener, the STAR Consortium, Sandra Nickel, Elisa Sittenberger, Lisa Schischke, Alina Geprägs, Isabell Liebhart, Andreas Witt, Cedric Sachser, Rebecca Brown, Vera Münch, Elisa König, Jörg Fegert, Ulrike Hoffmann, Inga Niedtfeld, Jenny Zähringer, Hasan-Hüseyin Isik, Sira Schug, Olaf Reis, Silvia Lindlar, Regina Waltes, Markus Mössner, Elisa Flach, Alexandra Edinger, Stephanie Bauer, Margarete Mattern, Sabine Herpertz

**Affiliations:** 1 Department for Child and Adolescent Psychiatry and Psychotherapy, Ulm University Medical Centre, Ulm, Germany; 2 Department of Psychiatry, Psychotherapy and Psychotraumatology Military Medical Centre, Ulm, Germany; 3 Department for Child and Adolescent Psychiatry and Psychotherapy, Rostock University Medical Centre, Rostock, Germany; 4 Department of Psychiatry and Psychotherapy III, Ulm University Medical Centre, Ulm, Germany; 5 Department of Child and Adolescent Psychiatry Psychosomatics and Psychotherapy, Goethe University Frankfurt, Frankfurt am Main, Germany; 6 Mental mHealth Lab, Institute of Sports and Sports Science, Karlsruhe Institute of Technology (KIT), Karlsruhe, Germany; 7 Department of Psychiatry and Psychotherapy, Medical Faculty Mannheim, Central Institute of Mental Health, Heidelberg University, Mannheim, Germany; 8 German Center for Mental Health (DZPG), Partner Site Mannheim, Mannheim, Germany; 9 Department for Child and Adolescent Psychiatry, Centre of Psychosocial Medicine, University Hospital Heidelberg, Heidelberg, Germany; 10 Department of Social Sciences, University of Mannheim, Mannheim, Germany; 11 University Hospital of Child and Adolescent Psychiatry and Psychotherapy, University of Bern, Bern, Switzerland; 12 German Center for Child and Adolescent Health (DZKJ), Partner Site Greifswald-Rostock, Rostock, Germany; 13 Faculty of Medicine and University Hospital Cologne, Department of Child and Adolescent Psychiatry, Psychosomatics and Psychotherapy, University of Cologne, Cologne, Germany; 14 Department of Behavioural and Cognitive Sciences, University of Luxembourg, Esch- sur-Alzette, Luxembourg; 15 Department of Psychosomatic Medicine and Psychotherapy, Central Institute of Mental Health Mannheim, Medical Faculty Mannheim, Mannheim University, Mannheim, Germany; 16 Department of Child and Adolescent Psychiatry, Medical University of Vienna, Vienna, Austria

**Keywords:** Nonsuicidal self-injury, Observational study, fMRI, Adolescents, Self-harm, Risk factors

## Abstract

**Background:**

Nonsuicidal self-injury (NSSI) is highly prevalent in adolescents and young adults worldwide. It is linked to a broad variety of mental disorders and an increased suicide risk. Despite its high prevalence, research on the underlying mechanisms and on potential risk and resilience factors for maintaining or quitting NSSI remains scarce. This manuscript presents an overview of the “Self-injury: Treatment-Assessment-Recovery” (STAR) collaboration, which aimed to address these gaps.

**Methods:**

We investigated the natural course of NSSI as well as its social, psychological, and neurobiological predictors (observational study; OS). OS data collection occurred at four timepoints (baseline [T0], 4 [post, T1], 12 [follow-up (FU), T2], and 18 [FU, T3] months after baseline) for the NSSI group, which was compared to a healthy control (HC) group at T0 only. Online self-report was used at all timepoints, while semi-structured interviews (face-to-face (f2f)) were conducted at T0 and T3. At T0 only, we conducted ecological momentary assessment and neurobiological investigations. Here, we present the general methodology and sample characteristics of the completed OS including the f2f subprojects, while other subprojects are not within the scope of this paper.

**Sample description:**

The OS sample consists of 343 participants at T0 (180 NSSI, 163 HC). Mean age in the NSSI group (T0) was 18.1 years (SD = 2.09, range: 15–25), gender-related data is available for 166: 156 = female, 7 = male, 3 = transgender, 10 = not disclosed). In the HC group, mean age (T0) was 19.1 years (SD = 2.35, range: 15–25) (142 = female, 21 = male). At T1, 128 (71.11%) of the NSSI participants completed the questionnaires, at T2 125 (69.44%) and at T3 104 (57.78%). In the fMRI subproject, 126 adolescents participated (NSSI = 66, HC = 60, 100% female; mean age (T0): NSSI = 18.10 years, SD = 2.21; HC = 19.08, SD = 2.36).

**Conclusion:**

Understanding predictors is of utmost importance for adequate diagnosis and intervention for NSSI. Our OS applied a multimodal investigation of social, psychological, and neurobiological parameters and is the largest sample of adolescents with NSSI to date including follow-up assessments. As health care providers require specific knowledge to develop new treatments, we believe that our in-depth assessments can potentially enhance care for youths engaging in NSSI.

## Background

Nonsuicidal self-injury (NSSI) is defined as deliberate, self-directed damage of body tissue without suicidal intent and for purposes not socially or culturally sanctioned [1]. According to the DSM-5, repetitive NSSI is classified as a distinct clinical phenomenon and a condition requiring further research [1]. Approximately one quarter to one third of the adolescents worldwide deliberately injure themselves at least once ( [2–4], and approximately 4% hurt themselves repetitively [5, 6]. Adolescents are more at risk for NSSI than adult populations: NSSI frequently starts at the age of 12 [2] and its prevalence peaks around the age of 15 to 16 years [7]. After that age, the frequency of NSSI decreases in most individuals, however, it can sometimes be replaced by other symptoms such as alcohol or substance misuse [7, 8].

NSSI poses a burden to the individuals affected as well as to their families. It is frequently linked to a variety of comorbid disorders, such as borderline personality disorder (BPD), depression, anxiety disorders, post-traumatic stress disorder (PTSD), substance abuse, and eating disorders [9, 10]. Adolescents engaging in NSSI have an increased risk for suicidal behaviour [11], however adolescents have improved suicide risk by discontinuing NSSI [12].

### Aetiology

A great body of research has focused on the functions and motivations of NSSI, with the main subcategories defined as an intrapersonal or self-regulating function (i.e. to control aversive emotional states) and an interpersonal or social function (i.e. communicating distress to others) [13]. Among those functions, emotion regulation seems to be the most stated motive [14]. However, NSSI frequently serves multiple purposes [9], which in combination contribute to its onset and maintenance. Thus, it is essential to identify these motives to contribute to the development of specific interventions.

Previous research has identified a multifactorial aetiological model based on biological, psychological, and social factors– the temporal framework model of NSSI. This recent model is based on the differentiation of trait and state markers [15]. Similar to biological models of other mental disorders, traits are characterised by rather stable, persisting behaviour and play a potentially causal role in the development of NSSI, or at least its predisposition. Traits can either be distal, such as vulnerabilities beginning around the time of birth, or they may have developed during a longer period of time. Or they can be proximal, which are “moderately stable but not expected to change within days or weeks” [15, p.230]. State markers represent the current status of being and are analysed to understand the preceding or subsequent conditions of NSSI. Here, ecological momentary assessments (EMA) have been highly useful in identifying predictors of NSSI, such as negative affect and the urge to self-injure [16]. More proximal biological risk factors include alterations in stress response systems, brain activation and pain processing, with certain biological states directly preceding or following NSSI and thus increasing the likelihood and reinforcing the behaviour [15]. However, it is important to note that this is a theoretical model based on neurobiological research with interdependent factors. Hitherto identified biological factors include (among others) a blunted cortisol response in social stressful situations, an increased pain threshold and pain tolerance, as well as alterations in emotion regulation processes involving a fronto-limbic circuitry [6, 17–19]. Genetic factors seem to play a less pronounced role than in other mental health problems (single nucleotide polymorphism (SNP) based heritability 13%) [20]. However, NSSI correlates with polygenic risk scores (PRS) for IQ (genetic correlation (rG) = 0.31) and is predicted by high PRS of ADHD, depression, and neuroticism [20].

Among the psychological factors, which are often reported regarding NSSI, are dysfunctional emotion regulation strategies, low stress tolerance, low self-esteem, and a highly self-critical attitude [21]. Social and environmental factors include weak communication skills, a lack of social support, peer victimization, dysfunctional family environment, adverse childhood experiences, NSSI within the peer group (peer contagion), and social media influence [22–26]. Social learning can shape the behaviours of adolescents at risk by showing that NSSI can be an effective coping strategy to reduce, for example, negative emotional states [27]. Particular attention has been given to social contagion via social media channels or the internet [24, 28, 29]. While often carefully hidden to peers and family, the anonymity of the internet seems to encourage sharing of NSSI experiences. However, online networking may also have a positive impact, as it offers the opportunity for affected people to exchange empathy, understanding and respect [30, 31] and encourages them to anonymously talk about NSSI with online friends [32], which may eventually lead affected youth to seek help. While previous research has covered these specific topics in different samples, a need to assess the interplay of the various factors in a large sample persists.

The “Self-injury: Treatment - Assessment - Recovery” (STAR) consortium was initiated as a collaborative project with research sites in the German cities of Ulm, Mannheim, Heidelberg, Landau, and Rostock and was funded by the German Federal Ministry of Education and Research. The aims of the overall project were to: (1) understand the natural course of NSSI in adolescents and young adults, (2) identify psychological, social, and neurobiological mechanisms and predictors of NSSI, (3) assess the effectiveness of an online intervention to reduce NSSI, and (4) provide a training approach for first response to NSSI in (mental) health care providers. The following sections inform about the general procedure and ethical aspects, followed by a detailed description of the subprojects.

### General study procedure of subprojects within the STAR project

**Fig. 1:**
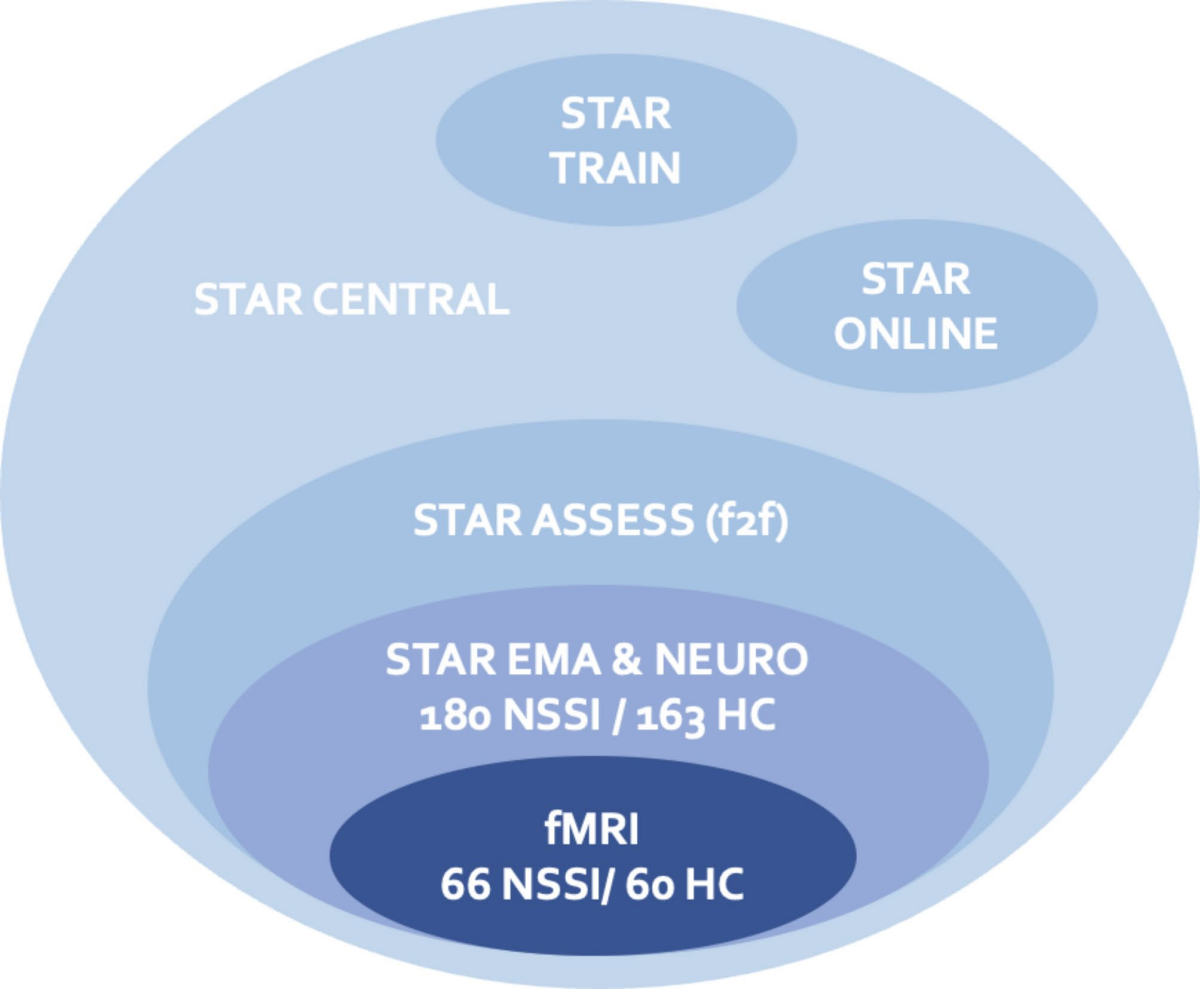
Overview of the subprojects. Numbers display the number of recruited participants in the subprojects. HC = healthy control group, NSSI = nonsuicidal self-injury group

To identify neurobiological, social, and psychological markers and to better understand the course of NSSI, six subprojects of the observation study (OS) were set up. STAR CENTRAL coordinated the communication between the centres. STAR ASSESS assessed the characteristics of a large sample of individuals with NSSI and healthy controls (HCs) by means of various questionnaires and clinical interviews with a focus on underlying psychological and social mechanisms and the aim to prospectively predict the development of NSSI courses. The entire sample was assessed online (STAR ASSESS (online part)). In addition, a subsample was also assessed face-to-face using clinical semi-structured interviews (STAR ASSESS face-to-face (f2f) part). The latter sample provided the basis for STAR NEURO, which aimed to investigate the genetic underpinnings of NSSI, biological parameters related to the autonomic nervous system (ANS) and the hypothalamic-pituitary-adrenal axis (HPA), as well as the perception of pain. STAR NEURO also investigated neurobiological markers using brain imaging in a subset of participants, who participated in a functional magnetic resonance imaging (fMRI) study. During the imaging task, participants performed an emotion regulation task and a social exclusion paradigm. An additional subproject based on the same sample as STAR NEURO was STAR EMA (ecological momentary assessments), which focussed on real-time assessments of various psychophysiological variables (e.g., cortisol, ECG) during a one-week assessment epoch in participants’ everyday lives (Fig. 1). A healthy control group was included for between group analyses of the neurobiological, social, and psychological markers described above.

STAR ONLINE focussed on the treatment of NSSI via an online approach and STAR TRAIN on the training of (mental) health care providers. The results of the STAR TRAIN subproject have been reported elsewhere, since they focused on a separate sample [33], while the general methodology of STAR ONLINE and ASSESS (online part) has been reported in a preregistration [34]. Thus, the present manuscript will focus on the recruitment of the OS and sample characteristics of the following subprojects: STAR ASSESS (f2f part), STAR NEURO, and STAR EMA.

## Methods

### Recruitment

Recruitment took place from the November 1st, 2017, until December 12th, 2023. Multiple paths of recruitment were used for the OS. Main sources of recruitment were social media platforms, such as Instagram, Facebook and Twitter/X, websites and online forums, including search-engine-based advertising via Google Ads. Additionally, flyers and posters were printed and distributed to clinical centres, outpatient settings, schools, and universities, with a focus on the surrounding area of the different centres. Further, for the NSSI sample, child and adolescent patients at the participating hospitals and outpatient clinics were directly invited to participate in the study. Registration for the project was centralised via the project website (https://star-projekt.de), where participants found information on the study and on NSSI. Inclusion criteria varied according to the subprojects. Inclusion criteria for HCs were participants age between 15 and 25 years, sufficient German language skills, no lifetime history of NSSI, no current mental disorder according to the DSM-5, and no current psychiatric or psychotherapeutic treatment. For inclusion in the online assessment of STAR ASSESS participants had to be between 15 and 25 years of age, have sufficient German language skills and report history of at least one NSSI incident in the last 12 months. To participate in STAR NEURO or STAR EMA and the accompanying f2f interviews, participants needed to report NSSI on five or more days within the last 12 months (criterion A in the DSM-5). To participate in the fMRI study, participants had to be able to travel to one of two available centres, in which fMRI scanning took place. Exclusion criteria for STAR NEURO and STAR EMA were substance or alcohol dependency of a severity to fulfil substance abuse criteria as defined in the DSM-5 within the last three months, pregnancy, epilepsy, acute suicidality that required immediate inpatient treatment, autism spectrum disorder, acute psychosis, or mental retardation. Specific exclusion criteria within the fMRI study included claustrophobia, metal parts in the body and a known history of brain alterations (e.g., tumour, epilepsy).

### Procedures

**Fig. 2:**
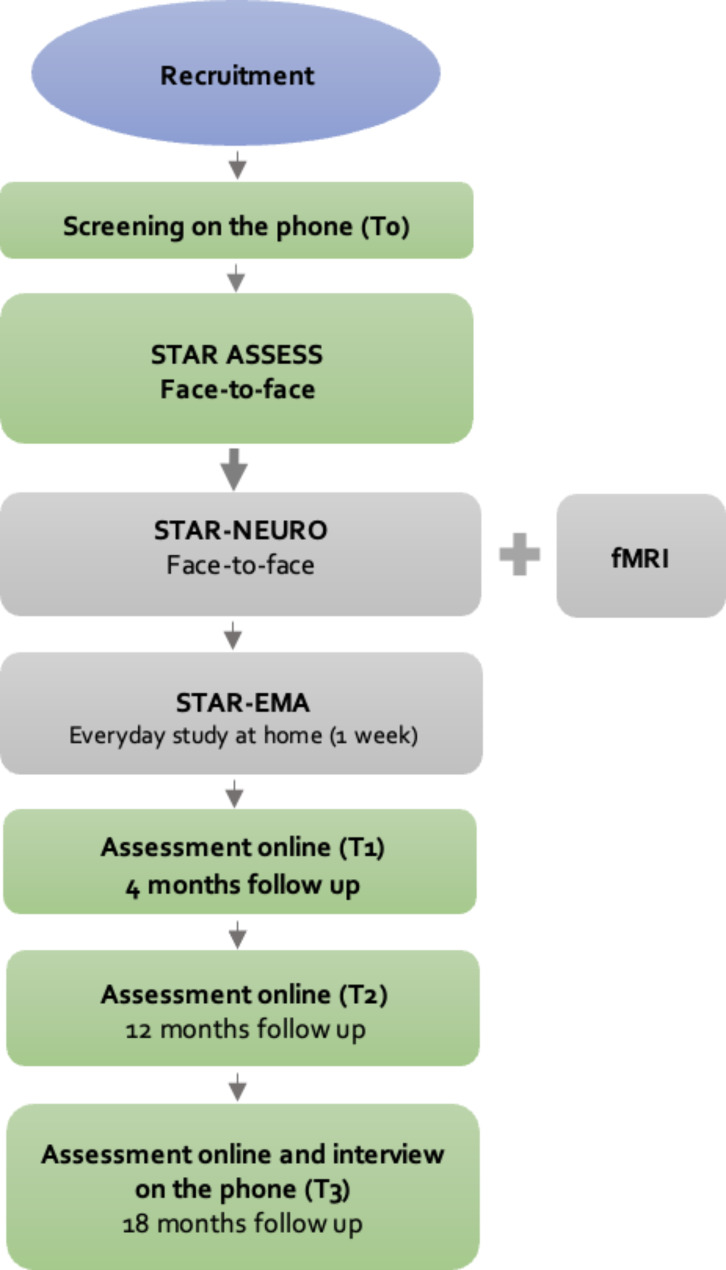
Flowchart of the participation process for the NSSI group for STAR NEURO, STAR EMA (ecological momentary assessments), and STAR ASSESS. Green rectangles: STAR ASSESS, grey rectangles: STAR NEURO and STAR EMA

After registration and written informed consent, psychopathology of the participants with NSSI was assessed online (see details below) at baseline (T0), 4 (T1), 12 (T2), and 18 (T3) months after the initial baseline assessment, to follow the course of NSSI and to assess potential predictors for NSSI (STAR ASSESS (online part)). In addition, participants were asked online whether they resided near one of the study centres, so that they could participate in the face-to-face assessment part of the STAR NEURO and STAR EMA study. If participants of the online assessment were eligible due to their place of living and agreed to participate in the f2f part, eligibility was further assessed via a telephone screening and informed consents were obtained. Within the f2f subsample, a smaller female sample was recruited for an fMRI study. In addition, the f2f subsample was followed up by phone at T3 in addition to the usual T3 online follow-up, in order to gain deeper insights into relevant variables. The HC group participated in STAR ASSESS face-to-face (f2f), STAR NEURO, and STAR EMA. In contrast to the NSSI group, the psychopathology of HCs was only assessed at baseline (T0). For a precise description of the participation process, please see Fig. 2. At T0 in- and exclusion criteria were checked.

### Ethical aspects

Study procedures for the STAR ASSESS (online part) were first reviewed by the Institutional Review Board (IRB) of the Medical Faculty of Heidelberg (University of Heidelberg), with recruiting centres also receiving approval from their respective IRB (University of Ulm, Medical Faculty of Mannheim, LEK Department of Psychology, University of Landau). Study procedures for the STAR ASSESS (f2f part), STAR NEURO and STAR EMA study were first reviewed by the IRB of the Medical Faculty Mannheim (University of Heidelberg), as the Mannheim centre took the lead for STAR NEURO, with recruiting centres also receiving approval from their respective IRB (University of Ulm, Medical Faculty of Heidelberg, LEK Department of Psychology, University of Landau). Participants with NSSI first provided online consent for the initial screening part, e.g. including questions regarding age and current NSSI, within STAR ASSESS (online part). For the further study procedure, participants and their caregivers provided written informed assent and consent. Healthy controls completed an online screening with information regarding age, lifetime NSSI, and current treatments. Afterwards, they received the participant information, had the telephone screening, and lastly, they and/or their parents provided written informed consent prior to the psychological f2f assessment via telephone. Like the NSSI group, informed consent was obtained for the further study procedure. For participants who completed the entire assessment, a compensation of 135€ was paid. In detail, 45€ were paid for the STAR NEURO procedure, 45€ for the fMRI scan, and 30€ for the STAR EMA participation. Additionally, in STAR EMA, participants were given a bonus of 15€, if they completed a minimum of 80% of the EMA prompts, wore the sensor and provided all cortisol samples (see *STAR EMA and NEURO daily measurements*). In case of dropouts during the diagnostic assessment, an hourly compensation of 10€/hour was paid.

The following sections present the foci of the separate subprojects.

### STAR ASSESS psychological assessment

To investigate various psychological predictors, a multi-method approach was used, including self-report questionnaires, an implicit measure, and clinical interviews. Several self-report questionnaires were used in the online assessment to assess sociodemographic data, psychopathology, NSSI severity, difficulties in emotion regulation, and self-efficacy, etc. Psychopathology in parents or other caregivers was investigated with another online questionnaire. For a detailed overview of the online measures see Table 1. Furthermore, exposure to potentially traumatic events, bullying, contagion effects of NSSI, and media consumption, especially related to NSSI, were assessed.

After the online assessment part, a psychological f2f assessment was conducted with the participants of the STAR EMA and STAR NEURO subsamples at T0. To assess the diagnostic criteria of major mental disorders according to the DSM-5, the Diagnostic Interview for Mental Disorders for adolescents (German: Jugendversion des Diagnostischen Interviews bei Psychischen Störungen, J-DIPS) [35], a structured interview, was used, which was modified for the STAR project. The modifications included the elimination of the section for bipolar disorder, gaming disorder, somatic stress disorders, additions related to NSSI from the Kinder-DIPS, and we abridged questions that were irrelevant for a diagnosis. Additionally, the J-DIPS includes questions to assess the research criteria of suicidal behaviour disorder (SBD) and of nonsuicidal self-injury (NSSI) as proposed in the DSM-5 Section III. The J-DIPS open access version with a detailed description of the adjustments made for the STAR project can be found online: (www.ruhr-uni-bochum.de/klipsychologie/dips-interv/kkjp/download/J-DIPS_OA_Gesamt.pdf). In addition, the Zanarini-Rating Scale for Borderline Personality Disorder (ZAN-BPD), with a modified time frame assessing six months instead of one week [36], the clinical global impression (CGI, severity scale), and the five-minute speech sample (FMSS) [37] to assess expressed emotions, were conducted. For the FMSS, the participants were asked to speak about their feelings and thoughts related to their parents or caregivers. The 18-month follow-up assessment of the STAR EMA & NEURO subsample included the J-DIPS, the ZAN-BPD, and the clinical global impression (CGI, severity, and improvement scale). For all assessments, the interviewers received an intensive standardised training.

**Table 1:** Online questionnaires and tasks assessed at T0, T1, T2, and T3

Questionnaire/Task	Assessed variables	Measurement timepoint			
		T0	T1	T2	T3
Kidscreen-10 [38]	Well-being and health-related quality of life	x	x	x	x
Patient health questionnaire-9 for adolescents (PHQ-A) [39]	Depressive symptomatology	x	x	x	x
NSSI severity questionnaire (NSSV-SG) [40]	NSSI severity	x	x	x	x
Paykel Suicide Scale (PSS) [41]	Suicidality in the past three months; lifetime suicidality	x	x	x	x
Standardised Assessment of Personality– Abbreviated Scale (SAPAS) [42]	Personality and potential personality disorders	x	x	x	x
Difficulties in Emotion Regulation Scale (DERS-18) [43]	Emotion regulation	x	x	x	x
Strengths and Difficulties Questionnaire (SDQ) [44]	Emotional and behavioural difficulties	x	x	x	x
General self-efficacy scale (SWE) [45]	General self-efficacy	x	-	-	x
Borderline Symptom List-23 (BSL-23) [46]	Self-report, quantitative assessment of Borderline-specific symptoms	x	x	x	x
Implicit association test (IAT) [47]	Implicit attitudes regarding NSSI	x	-	-	x
Childhood trauma questionnaire short form (CTQ-SF) [48]	Impact of adverse lifetime experiences	x	-	-	-
Brief Symptom Inventory-53 (BSI-53) [49, 50]	Psychological stress in parents/caregiver of the participants	x	-	-	-

T0 = Baseline assessment, T1 = 120 days after baseline, T2 = follow-up 360 days after T0, T3: FU 2 = follow-up 540 days after T0

### STAR NEURO laboratory assessment

#### Blade paradigm, genetic analyses, and analysis of the peripheral stress response systems

First, the investigator took a saliva sample for DNA extraction from the participants. Genetic analysis included SNP-Microarray analysis (Illumina Human GSA + Psych Bead Array v4.0) to calculate polygenic risk scores for ADHD, ASD, depression, anxiety, neuroticism [20], to perform quantitative GWAS integrating brain developmental transcriptome data [51], and to explore artificial intelligence (AI)-driven pathways based molecular burden scores as predictors for NSSI-diagnosis and course as well as biological markers such as cortisol reaction levels [52].

**Fig. 3:**
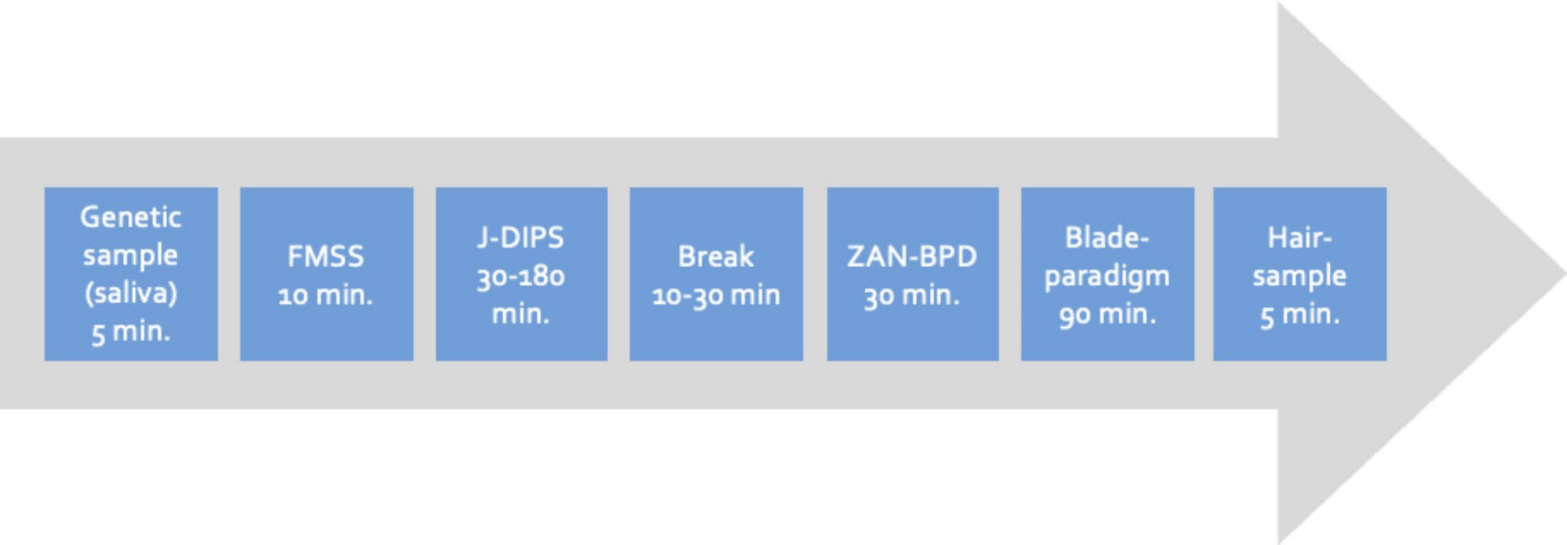
f2f assessment at the centres. FMSS = five-minute speech sample, J-DIPS = German: Jugendversion des Diagnostischen Interviews bei Psychischen Störungen, ZAN-BPD = Zanarini-Rating Scale for Borderline Personality Disorder

To investigate the effect of NSSI on cortisol levels and electrocardiogram (ECG), participants underwent the blade paradigm [53], which by means of a weighed blade allows the simulation of “cutting pain” without any damage to the skin tissue. ECG was continuously recorded at 1024 Hz with an EcgMove 3 sensor (Movisens GmbH; Karlsruhe, Germany), attached to a chest belt with dry electrodes in order to continuously assess heart rate (HR) and HR-variability (HRV). Salivary cortisol was assessed 7 times every 10–15 min starting 25 min before the blade-paradigm and continuing throughout the experiment by chewing on a cotton swap (Salivette^®^; Sarstedt, Numbrecht, Germany) for one minute respectively. Samples were frozen at -20 °C until assay. In addition, participants rated the perceived pain intensity on a smartphone using a visual analogue scale. Lastly, the investigator cut a thin strand of hair from the back of the head, as close to the scalp as possible for interindividual cortisol analyses. For the analyses of baseline cortisol levels, only the last three centimetres were analysed. Both saliva and hair cortisol samples were analysed at the Biopsychology Laboratory at the Technical University of Dresden. The entire assessment had a duration of approximately five hours and is visualised in Fig. 3.

### STAR NEURO fMRI

Participants at the centres Ulm and Mannheim were asked to participate in a functional magnetic resonance imaging study (fMRI). This sub-study focused on emotion regulation and social exclusion. Investigating the neural correlates of emotion regulation, participants were presented with unpleasant or neutral images of a subset of 80 pictures of the IAPS [54] partly coupled with an unpleasant heat stimulus by means of an fMRI-compatible thermode (Ulm: ATS-Thermode, 30 × 30 mm, TSA-II, Medoc Advanced Medical Systems, Ramat Yishai, Israel; Mannheim: CHEPS-Pathway, 30 × 30 mm, Medoc Advanced Medical Systems, Ramat Yishai, Israel). In a second step, they participated in the Cyberball paradigm to evoke social exclusion [55]. Cyberball is a game with three conditions, where the participant is instructed to either observe or participate in a ballgame. The participation conditions either include or exclude the participant from the game, leading to feelings of social inclusion or rejection. Acquisition of magnetic resonance imaging data was performed on a 3 Tesla MAGNETOM Prisma (Siemens AG, Erlangen, Germany) with a 64-channel head/neck coil. The fMRI scan was concluded with another set of questionnaires regarding social exclusion and rating of the IAPS images. Differences between the scanners were statistically controlled for by the study centres.

### STAR EMA and NEURO daily measurements

**Fig. 4:**
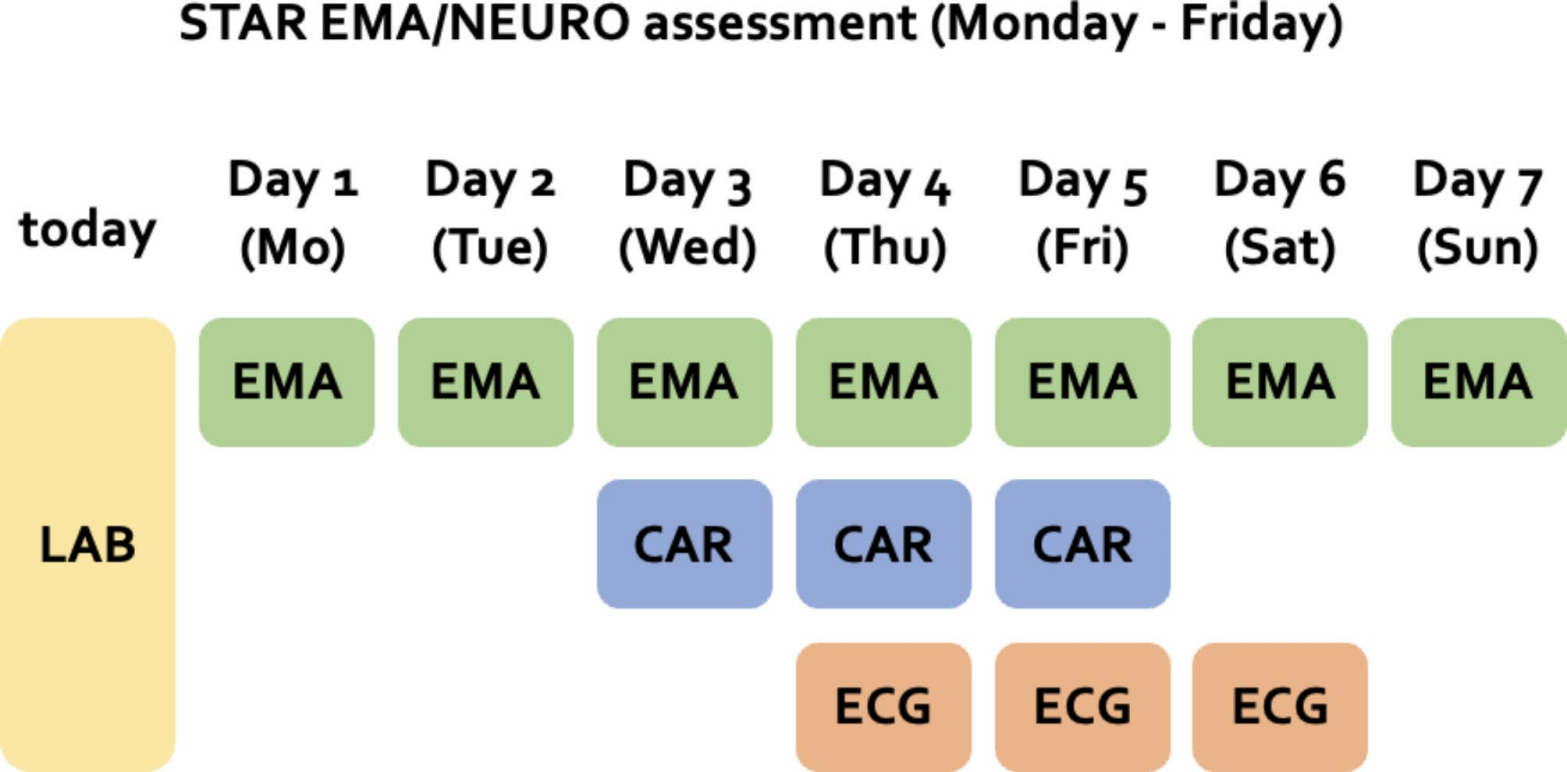
STAR EMA/NEURO assessment. LAB: f2f assessment in the laboratory; EMA (ecological momentary assessments): assessments via smartphone during participants’ daily lives; CAR: Cortisol awakening response, saliva sampling in the morning and evening; ECG: wearing of the ECG belt from Thursday evening until Saturday evening

Participants received a study smartphone, which they carried for seven days from Monday to Sunday while going about their everyday lives. The smartphones were programmed using movisensXS (movisens GmbH, Karlsruhe, Germany) to elicit prompts according to an individualized time-based sampling scheme. Participants chose a wake-up time for each of the seven days of the assessment and specified their timetable to only receive prompts in their free time (e.g., after school hours) during weekdays. Thus, participants were asked to answer one assessment in the morning (i.e., the morning assessment) plus hourly assessments during the predefined assessment interval (i.e., repeated assessment every 60 min +/- 10 min from the individualized starting point through 22:00). At the weekends, participants were prompted from their individualised wake-up time until 22:00. The prompted questionnaires measured momentary mechanisms regarding NSSI behaviour. They included morning assessments regarding sleep quality (4 items, 53) and repeated assessments with 25 items regarding affect (valence and arousal with 2 items each, based on the Multidimensional Mood Questionnaire [57], and the intensity of six specific emotions (e.g., shame, self-contempt and anger, with 1 item each), occurrences of NSSI (i.e., acts and urges, 1 item each), dissociative symptoms (4 items [58]),, interpersonal behaviour (2 items), impulsivity (1 item), stress-reactivity and reward experience (1 item each), and momentary self-esteem (4 items, based on [59]). Answering the repeated prompts usually took participants less than one minute. During the respective week of the EMA assessment, participants were also instructed to wear an ECG belt (see above for details) for 48 h from Thursday evening 8 p.m. to Saturday evening 8 p.m. Further, participants provided 4 daily saliva samples on 3 days to quantify the cortisol awakening response (CAR, 3 samples following awakening) in the morning and the diurnal slope in cortisol secretion (additional sample in the evening). Saliva samples were registered with the study smartphones to enable accurate tracking of sampling (see Fig. 4). On the days before the cortisol assessments, participants received an additional set of questions regarding stress anticipation (4 items [60]), in the evenings. All STAR EMA participants are also part of the STAR NEURO subsample and provided additional data on potential confounding variables of interest concerning biological samples (e.g., hormonal contraceptives, general medication intake and regular intake of medication containing glucocorticoids, physical illness).

## Sample description

### Demographic characteristics

#### STAR ASSESS (f2f part), STAR NEURO & STAR EMA

The sample for the subprojects STAR ASSESS (f2f part), STAR NEURO, and STAR EMA consists of *N* = 343 participants (NSSI sample *n* = 180, healthy controls (HC) *n* = 163). In the NSSI group, gender-related data is available for 166 participants (156 = female, 7 = male, 3 = transgender, 10 = not disclosed). Mean age in the NSSI group was 18.1 years (SD = 2.09, range: 15–25). Regarding the STAR EMA and STAR NEURO sample, 114 (63.3%) underwent psychotherapeutic treatment at the time of the study (participants could select multiple options; day hospital *n* = 35, in-patient treatment: *n* = 79, out-patient treatment: *n* = 115, health centres: *n* = 20, psychopharmacotherapeutic treatment: *n* = 67). In the HC group (142 = female, 21 = male), mean age was 19.1 years (SD = 2.35, range: 15–25). Mean age between the HC and the NSSI group differed significantly (*p* < 0.001). School/education related data are presented in Table 2.

In the NSSI group, regarding FU data, at T1, 128 participants (71.11%) completed the FU questionnaires, at T2 125 (69.44%) and at T3 104 (57.78%).

Of the total f2f sample, *n* = 126 participated in the fMRI study (NSSI = 66, HC = 60). In the fMRI subproject, NSSI participants were all female and mean age was 18.10 years (SD = 2.21). In the healthy control group for the fMRI subsample, participants were all female, with an average age of 19.08 (SD = 2.36).

**Table 2:** Data on education, school, and work for the NSSI and the HC groups of STAR ASSESS (f2f part), NEURO, and EMA sample

Item	NSSI (*n* = 162)	HC (*n* = 163)
**I am currently…** At school	80	53
An apprentice	39	84
Employed	13	19
Seeking work	30	7
**School degree** Not graduated	2	0
Secondary modern school (German: Hauptschule, school year 5–9)	13	1
Secondary school (German: Realschule, school year 5–10)	32	3
A-levels (German: Abitur, schoolyear 5–12/13 depending on the state)	35	106
**Current or last school attended**		
School for children with special needs” (German: Förderschule)	1	0
Secondary modern school (German: Hauptschule/Realschule Plus)	10	1
Comprehensive school (German: Gesamtschule)	16	6
Secondary school (German: Realschule)	30	6
Grammar school (German: Gymnasium)	74	131
Other	31	18

HC = healthy control group, NSSI = nonsuicidal self-injury group

## Discussion

The STAR subprojects STAR ASSESS (f2f), STAR EMA and STAR NEURO have collected data from 343 subjects (180 with repetitive NSSI and 163 healthy controls) at seven sites in Germany (Heidelberg, Landau, Karlsruhe, Mannheim, Neuruppin, Ulm, Rostock). In addition, data on these individuals was complemented by data from the online assessment of the larger STAR ASSESS OS sample. The STAR project aims to thoroughly assess psychological, social, neurobiological data and their interplay that allow a variety of analytic approaches. Focusing on the developmental period of adolescence, the age range of the overall sample was between 15 and 25 years and the sample was recruited from several sites, such as schools, via social media, and from treatment settings. We included females, males and transgender individuals, but most of the participants are female, which is in line with epidemiological data on NSSI, reporting higher prevalence rates of NSSI in females [2]. However, this may limit the generalizability of the results, which may further be restricted to participants that were fluent in German. To the best of our knowledge, the recruited STAR EMA and STAR NEURO sample of 343 adolescents, providing information on parameters, such as EMA in everyday live, laboratory data on cortisol and genetics as well as fMRI data (in a subsample), constitutes the world-wide largest sample of in-depth neurobiologically characterised adolescents engaging in NSSI to date. Furthermore, the comprehensive data might be able to shed light on the development of other mental disorders, such as BPD, given that a wide range of psychological parameters were assessed, which will allow further analyses. The acquired information will allow us to evaluate predictors and risk factors, and hopefully allow future researchers to tailor therapeutic offers. Moreover, it provides us with the opportunity to link multiple markers of interest and gain a more holistic picture of NSSI.

### Strengths and limitations

We applied a multimodal investigation of social, psychological, and neurobiological parameters and to date we present the largest sample of adolescents with NSSI including follow-up assessments in females, males and transgender. However, it is rather homogenous regarding the gender distribution. Future studies may incorporate a more heterogenous sample, however, we did not selectively opt for the sample distribution as given. One of the reasons why adolescents with other cultural backgrounds did not participate in this study could be found in language or cultural barriers with regard to seeking help for mental health problems [61] or potentially also for participating in research [62].

## Data Availability

Data and material can be shared upon individual request to the authors.
